# Lectures in the Medical College of Ohio

**Published:** 1828-07

**Authors:** 


WESTERN MEDICAL LECTURES.

17. *Medical College of Ohio.*—The Lectures of this Institution will commence on the first Monday in next November; and will continue without intermission, till the end of the ensuing February, on the following subjects, viz:—

*Anatomy and Physiology*, by JEDEDIAH COBB, M. D.
*Materia Medica*, by CHARLES E. PIERSON, M. D.
*Surgery*, by JESSE SMITH, M. D.
*Chemistry and Pharmacy*, by Rev. ELIJAH SLACK, M. D.
*Obstetricks and Diseases of Women and Children*, by JOSIAH WHITEMAN, M. D.
*Theory and Practice of Medicine*, by JOHN MOORHEAD, M. D.

The price of the Anatomical Ticket is $12; that of each of the others is $10.

The fee for Matriculation, including the use of the Library, and admittance to the Hospital, is $3. In the Hospital, (the Commercial Hospital and Lunatic Asylum of Ohio) a regular course of Clinical instruction is delivered on the most important of the cases presented.

The cost of graduation is $21, being three dollars to each Professor and three for the Diploma. The Commencement for conferring degrees, is held immediately after the close of the Examination at the end of the Session.

From its connexion with the Commercial Hospital and Lunatic Asylum of Ohio, this Institution furnishes, beyond question, such opportunities of acquiring anatomical knowledge, as cannot be *surpassed* elsewhere in the Western country.

By order of the Faculty,

JEDEDIAH COBB, DEAN.

*Medical College of Ohio, Cincinnati,*
*July 9th*, 1828.

